# Neuroharmony: A new tool for harmonizing volumetric MRI data from unseen scanners

**DOI:** 10.1016/j.neuroimage.2020.117127

**Published:** 2020-10-15

**Authors:** Rafael Garcia-Dias, Cristina Scarpazza, Lea Baecker, Sandra Vieira, Walter H.L. Pinaya, Aiden Corvin, Alberto Redolfi, Barnaby Nelson, Benedicto Crespo-Facorro, Colm McDonald, Diana Tordesillas-Gutiérrez, Dara Cannon, David Mothersill, Dennis Hernaus, Derek Morris, Esther Setien-Suero, Gary Donohoe, Giovanni Frisoni, Giulia Tronchin, João Sato, Machteld Marcelis, Matthew Kempton, Neeltje E.M. van Haren, Oliver Gruber, Patrick McGorry, Paul Amminger, Philip McGuire, Qiyong Gong, René S. Kahn, Rosa Ayesa-Arriola, Therese van Amelsvoort, Victor Ortiz-García de la Foz, Vince Calhoun, Wiepke Cahn, Andrea Mechelli

**Affiliations:** a Department of Psychosis Studies, Institute of Psychiatry, Psychology & Neuroscience, King’s College London, 16 De Crespigny Park, SE5 8AF, United Kingdom; b Department of General Psychology, University of Padova, Via Venezia 8, Padova, Italy; c Center of Mathematics, Computing, and Cognition, Universidade Federal do ABC, Santo André, Brazil; d Department of Psychiatry, School of Medicine, Trinity College Dublin, Dublin, Ireland; e Laboratory of Neuroinformatics, IRCCS Istituto Centro San Giovanni di Dio Fatebenefratelli, Brescia, Italy; f Orygen, The National Centre of Excellence in Youth Mental Health, University of Melbourne, Melbourne, Victoria, Australia; g Centre for Youth Mental Health, University of Melbourne, Melbourne, Victoria, Australia; h Centro Investigación Biomédica en Red de Salud Mental (CIBERSAM), Spain; i Departamento de Psiquiatria, Universidad de Sevilla, Instituto de Biomedicina de Sevilla (IBIS), Spain; j Hospital Universitario Virgen del Rocío, Sevilla, Spain; k Department of Psychiatry, Marqués de Valdecilla University Hospital, IDIVAL, School of Medicine, University of Cantabria, Santander, Spain; l Clinical Neuroimaging Laboratory, School of Medicine & Center for Neuroimaging and Cognitive Genomics, NUI Galway University, Galway, Ireland; m Neuroimaging Unit, Technological Facilities, Valdecilla Biomedical Research Institute IDIVAL, Spain; n School of Psychology & Center for Neuroimaging and Cognitive Genomics, NUI Galway University, Galway, Ireland; o Department of Psychiatry and Neuropsychology, School of Mental Health and Neuroscience, Maastricht, the Netherlands; p Discipline of Biochemistry & Center for Neuroimaging and Cognitive Genomics, NUI Galway University, Galway, Ireland; q Memory Clinic and LANVIE-Laboratory of Neuroimaging of Ageing, University Hospitals and University of Geneva, Geneva, Switzerland; r Laboratory of Alzheimer’s Neuroimaging and Epidemiology - LANE, IRCCS Istituto Centro San Giovanni di Dio Fatebenefratelli, Brescia, Italy; s State University, Georgia Institute of Technology, Emory University, Atlanta, GA, USA; t Department of Child and Adolescent Psychiatry/Psychology, Erasmus Medical Centre - Sophia Children’s Hospital, Rotterdam, Netherlands; u Section for Experimental Psychopathology and Neuroimaging, Department of General Psychiatry, Heidelberg University, Germany; v Center for Translational Research in Systems Neuroscience and Psychiatry, Department of Psychiatry and Psychotherapy, University Medical Center Göttingen, Germany; w Huaxi MR Research Center (HMRRC), Department of Radiology, West China Hospital of Sichuan University, Chengdu, China; x Psychoradiology Research Unit of Chinese Academy of Medical Sciences, West China Hospital of Sichuan University, Chengdu, Sichuan, China; y Department of Radiology, Shengjing Hospital of China Medical University, Shenyang, Liaoning, China; z Department of Psychiatry, University Medical Center Utrecht Brain Center, Utrecht, the Netherlands; aa Department of Psychiatry, Icahn School of Medicine at Mount Sinai, New York, NY, USA; ab Tri-institutional Center for Translational Research in Neuroimaging and Data Science (TReNDS), Georgia

## Abstract

• We present Neuroharmony, a harmonization tool for images from unseen scanners.
• We developed Neuroharmony using a total of 15,026 sMRI images.
• The tool was able to reduce scanner-related bias from unseen scans.
• Neuroharmony represents a significant step towards imaging-based clinical tools.
• Neuroharmony is available at https://github.com/garciadias/Neuroharmony.

The increasing availability of magnetic resonance imaging (MRI) datasets is boosting the interest in the application of machine learning in neuroimaging. A key challenge to the development of reliable machine learning models, and their translational implementation in real-word clinical practice, is the integration of datasets collected using different scanners. Current approaches for harmonizing multi-scanner data, such as the ComBat method, require a statistically representative sample; therefore, these approaches are not suitable for machine learning models aimed at clinical translation where the focus is on the assessment of individual scans from previously unseen scanners. To overcome this challenge, we developed a tool (‘Neuroharmony’) that is capable of harmonizing single images from unseen/unknown scanners based on a set of image quality metrics, i.e. intrinsic characteristics which can be extracted from individual images without requiring a statistically representative sample. The tool was developed using a mega-dataset of neuroanatomical data from 15,026 healthy subjects to train a machine learning model that captures the relationship between image quality metrics and the relative volume corrections for each region of the brain prescribed by the ComBat method. The tool resulted to be effective in reducing systematic scanner-related bias from new individual images taken from unseen scanners without requiring any specifications about the image acquisition. Our approach represents a significant step forward in the quest to develop reliable imaging-based clinical tools. Neuroharmony and the instructions on how to use it are available at https://github.com/garciadias/Neuroharmony.

## 1 Introduction

Over the past few years, neuroimaging research has shifted its focus from group level to individual level analysis, with the ultimate aim of generating results that can be translated into clinical practice. In particular, the constantly growing number, size, and availability of MRI research datasets in the last decades (e.g., Jack et al., 2008; Miller et al., 2016; Thompson et al., 2017) has boosted interest in the application of machine learning methods to the investigation of brain disorders (Arbabshirani et al., 2017; Lemm et al., 2011; Ma et al., 2018; Vieira et al., 2017). A number of successful applications to brain disorders have been reported including, for example, Alzheimer’s Disease (AD) (Gerardin et al., 2009), depression and mood disorders (Nouretdinov et al., 2011), autism (Ecker et al., 2010) and schizophrenia (Lei et al., 2019). Yet, translational implementation in the real word remains limited (Focke et al., 2011; Orrù et al., 2012). An important challenge to such implementation is the use of different MRI scanners and acquisition protocols resulting in inconsistent measures of brain region volumes (Clark et al., 2006; Han et al., 2006; Jovicich et al., 2006; Lee et al., 2019; Takao et al., 2011). In particular, inconsistencies can arise from the MRI machine field strength, head motion, gradient non-linearity, time-of-day, among others (Goto et al., 2012; Keshavan et al., 2016; Krueger et al., 2012; Lee et al., 2019; Takao et al., 2011; Trefler et al., 2016). A number of multi-scanner studies have adopted a rigid acquisition protocol, including phantom calibration (Maikusa et al., 2013) to mitigate these inconsistencies. However, this requires a priori coordination with regards to the image acquisition protocol between the different centers and it therefore is not an option if the aim is to leverage already existing data.

In order to mitigate scanner and protocol effects, various data harmonization methods have been proposed (Doran et al., 2005; J. P. Fortin et al., 2018; J. P. Fortin et al., 2017; Jovicich et al., 2006; Keshavan et al., 2016; Maikusa et al., 2013). Data harmonization consists of performing calibration corrections to data from different sources with the aim of making their comparison more meaningful. The aim of the harmonization process is not necessarily to approximate the measurements to the ground truth (i.e., the real volume of brain regions) but to make the comparisons among subjects more reliable at both the individual and group level. Therefore, harmonization does not eliminate possible systematic bias, but guarantees that the distortion affects all data points in the same way. For instance, the ComBat harmonization tool (J. P. Fortin et al., 2018; J. P. Fortin et al., 2017; Johnson et al., 2007) uses Bayesian regression to find systematic differences among multiple data collected using different scanners. The tool performs additive and multiplicative corrections to produce distortions that eliminate these systematic differences from the data. The main limitation of this approach is the need for a sample size that guarantees a statistically representative sample from each scanner included in the study. This presents a challenge for the translational implementation of machine learning models in clinical practice. To be useful in real world clinical practice, a trained model must be able to make predictions about a single image from a scanner that was not part of the training set. In other words, the model must be able to extrapolate the features to unseen data from unknown scanners in the absence of a statistically representative sample from each scanner. It follows that existing harmonization tools, such as ComBat, are not suitable for machine learning models aimed at clinical translation. In order to address this challenge, we need harmonization procedures that do not require a statistically representative sample for each scanner. Ideally, a flexible machine learning-based tool would require no a priori calibration of the images and no information about the MRI scanner and protocol. In this paper, we developed a tool that takes a first step in this direction.

In particular, we propose a new approach to harmonization that does not require a statistically representative sample for each scanner and protocol. Tardif et al. (2009) showed that contrast-to-noise ratio impacts the results of voxel-based morphometry studies. Following this observation, Esteban and colleagues developed a series of image quality metrics (IQMs) to perform quality control of MRI images in multiple datasets (Esteban et al., 2017, 2019). These metrics - which include contrast-to-noise ratio and other intrinsic characteristics - are directly measurable from individual MRI images without requiring a statistically representative sample. Critically, IQMs were shown to be associated with the scanner used to acquire the images. For example, the contrast between grey matter (GM) and white matter (WM) was found to vary strongly between different acquisitions protocols and scanners (Esteban et al., 2017). Based on these background findings, we hypothesized that the use of these intrinsic characteristics of the images could be used to aid data harmonization. In order to test this hypothesis, we first evaluated the ComBat harmonization tool (J. P. Fortin et al., 2018; J. P. Fortin et al., 2017; Johnson et al., 2007) using a mega-dataset comprising a total of 15,026 structural neuroanatomical scans from healthy subjects from 62 scanners. This evaluation showed that ComBat was able to reduce scanner-related differences as expected. We then trained a machine learning tool (‘Neuroharmony’) that captured the relationship between the IQMs and the corrections to the relative volumes of each region of interest (ROI) prescribed by the ComBat harmonization. Finally, we applied Neuroharmony to images from unseen scanners to predict the relative volume corrections showing its ability to reduce variation in the data due to inter-scanner variability. To our knowledge, Neuroharmony is the first tool capable of harmonizing single images from unseen datasets.

## 2 Material and methods

### 2.1 Datasets

**Fig. 1:**
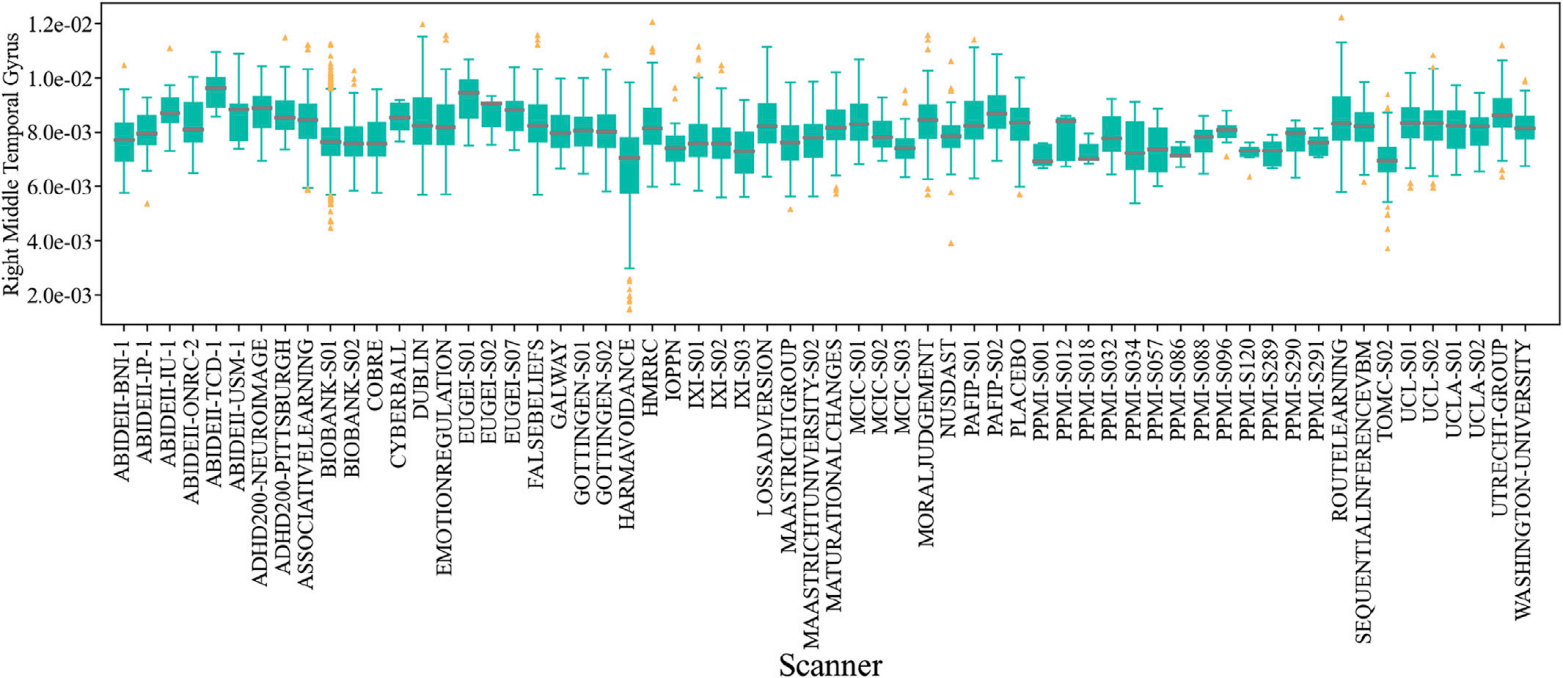
Box plot of the right middle temporal gyrus relative volumes for all scanners included in our study. A grey horizontal line marks the median value in each dataset, the solid green boxes present the inter-quantile ratio in each dataset. The vertical green lines cover 90% of the measurements in each dataset. The yellow triangles represent data points outside the 5–95% interval.

The initial sample of our study included 18,190 T1-weighted MRI images of healthy controls from 89 scanners. We excluded all subjects younger than 18 years old and older than 70 years old. Upon visual inspection, we observed that some of the images were affected by motion, poor contrast-to-noise ratio or other artifacts. To exclude poor quality images, we used the MRIQC^1^ tool with the standard parameters (Esteban et al., 2017). This tool uses 68 IQMs to determine the probability of an image being unusable. We discarded any image where this probability was higher than 0.5. We also excluded all outliers with regards to relative brain volume measurements, since outliers are unexpected in healthy subjects and are likely to be due to artifacts resulting from poor segmentation. A subject was considered an outlier if the relative volumes of at least 10 regions of interest (ROIs), corresponding to ~10% of the feature space, were more than 2.5 standard deviations (σ) away from the sample mean (μ). Here ‘relative volume’ refers to the volume of each ROI divided by the total intracranial volume of the subject. We iteratively repeated this process, recalculating μ and σ until no additional subject met our criteria for being an outlier. This process was implemented within each scanner, in order to ensure that subjects would not be considered outliers simply because of differences among scanners. To ensure the quality of the FreeSurfer preprocessing (described below) we applied the Qoala quality control tool (Klapwijk et al., 2019) excluding any image with a probability of not being usable higher than 0.5. After excluding images of poor quality, outliers, and subjects with any missing data, we selected all scanners available with enough statistical representation, for which we defined a threshold of 5 subjects per scanner (based on Fortin et al., 2018 showing that the algorithm works for samples as small as 5 subjects). The final sample comprised of 15,026 subjects from 62 scanners on 32 datasets, ABIDEII (Nielsen et al., 2013), ADHD200^2^ (Milham et al., 2012), ASSOCIATIVE LEARNING (Bursley et al., 2016), BIOBANK (Miller et al., 2016), COBRE (Çetin et al., 2014), CYBERBALL (Romaniuk et al., 2016), DUBLIN, EMOTION REGULATION (Wager et al., 2008), EU GEI, FALSE BELIEFS (Moran et al., 2012), GALWAY, GOTTINGEN, HARM AVOIDANCE (Van Schuerbeek et al., 2016), HMRRC, IOPPN (Benetti et al., 2013), IXI (Heckemann et al., 2011), LOSS AVERSION, MAASTRICHT UNIVERSITY, MAASTRICHT GROUP, MATURATIONAL CHANGES (Velanova et al., 2008), MCIC^3^ (Gollub et al., 2013), MORAL JUDGMENT (Chakroff et al., 2016), NUSDAST (Wang et al., 2013), PLACEBO (Tétreault et al., 2016), PPMI^4^ (Marek et al., 2011), ROUTE LEARNING (Chanales et al., 2017), PAFIP (Pelayo-Terán et al., 2008), SEQUENTIAL INFERENCE VBM (FitzGerald et al., 2017), TOMC (Frisoni et al., 2009), UCL, UCLA (Poldrack et al., 2016), UTRECHT GROUP, WASHINGTON UNIVERSITY (Power et al., 2015). A table with detailed information for all included scanners can be found in the supplementary material,^5^. Fig. 1 shows the distribution of the relative volume of the right middle temporal gyrus for all the included scanners; this region was chosen as a typical example to illustrate the variations found across the different scanners. It can be seen that the distribution varied substantially across scanners.

The collection of all data was approved by the local ethics committees. Informed consent, including the sharing of the images for future research, was obtained from all participants.

### 2.2 Preprocessing

All T1-weighted images were preprocessed using the recon-all function from the FreeSurfer package version 6.0.0^6^ (Fischl et al., 2002) with the standard parameters. In this case, FreeSurfer sets the same random seed to all runs and stochastic effects of the reconstruction is consistent among subjects. For each image, the volumes of 101 ROIs were extracted and normalized by dividing the volume of each region by the total intracranial volume of the subject (see supplementary material for the complete list of ROIs). These regions were extracted based on the Desikan-Killiany atlas (Desikan et al., 2006) and on the ASEG atlas (Fischl et al., 2002).

### 2.3 Demographics

**Fig. 2:**
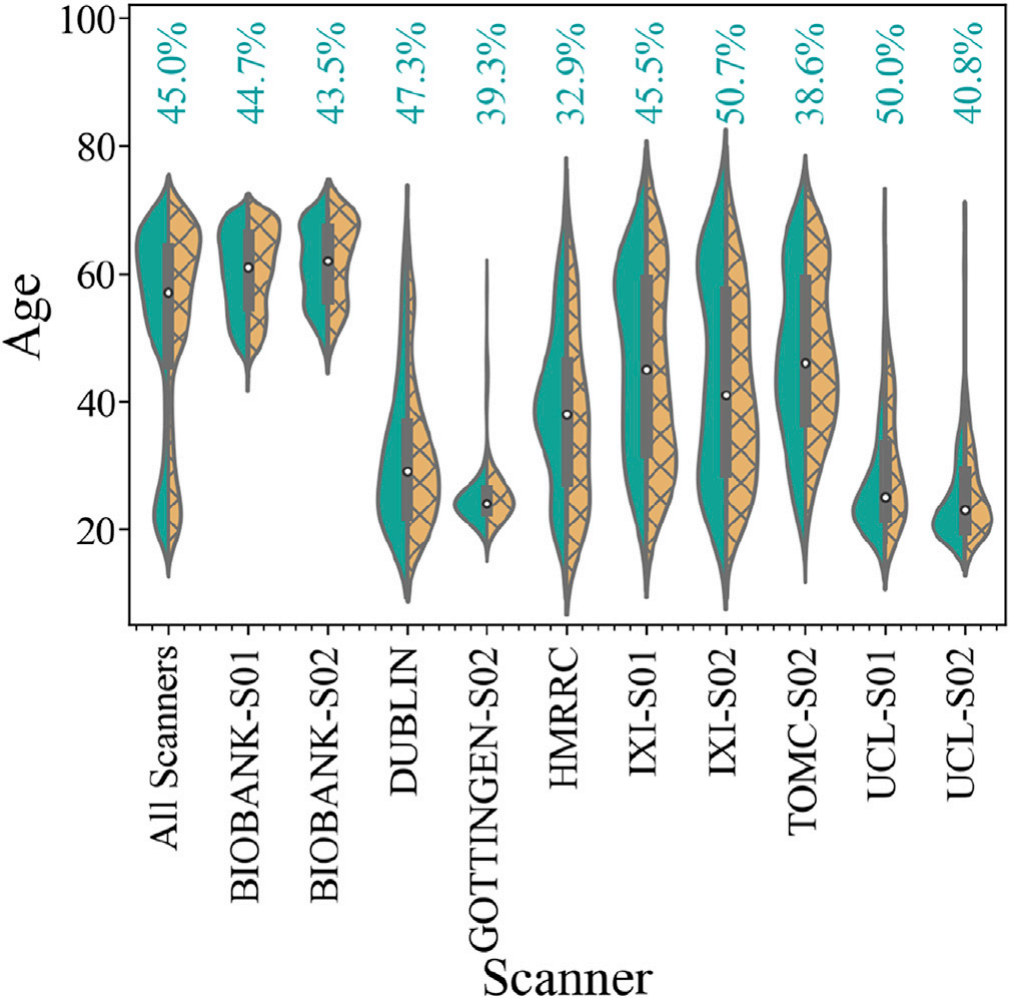
Violin plot showing age distribution for male (in green, left distribution) and female (in yellow, right distribution) subjects for all datasets along with the individual distribution of the 10 largest scanner samples.

**Fig. 3:**
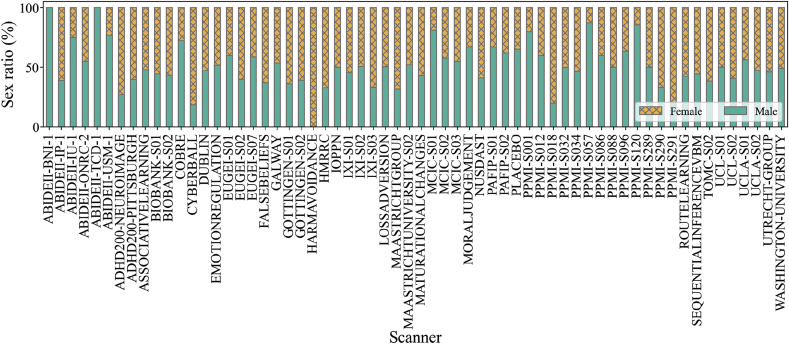
The ratio of subjects of each sex in the data from all included scanners. The plot shows bars corresponding to 100% of the subjects measured with each scanner. The portion of male subjects is colored in green and the portion of female subjects is colored in yellow and X-hatched.

The sample from each scanner used in this study covered a broad range of ages. Overall, the data from each scanner were highly unbalanced in terms of age and sex, as shown in Fig. 2 and Fig. 3. In the whole dataset, 55% of the subjects were female. Fig. 2 shows the distribution of ages for male and female subjects in 10 of the largest scanners, while Fig. 3 shows the sex ratios for all scanners. It is evident that some of the scanners only contained subjects of one sex. We can also see that there is almost no overlap in the age range between certain pairs of scanners. Considering these differences, we assessed scanner bias after taking the effects of sex and age into account (below). As detailed in the supplementary material, different scanners often used different acquisition protocols. In this article, we use the expression “scanner bias” regardless of the overlap between acquisition protocols. However, it is important to stress that both scanner and acquisition protocol can affect the quality of the images and the measure of volumes.

### 2.4 ComBat harmonization

Fortin et al. (2018) compared three types of scanner harmonization, which they called *residual, adjusted residual* and *ComBat* harmonization. From these methods, the most robust results were achieved by the ComBat harmonization. This procedure consists of performing a Bayesian regression that corrects the measurements from different samples with additive and multiplicative terms. The complete description of the model can be found in Johnson et al. (2007).

In this study, we used the python version of the ComBat software that can be found at https://github.com/ncullen93/neuroCombat. The harmonization process was done over the relative ROI volumes.

The ComBat tool performs the harmonization based on a given covariate while conserving the variance due to other covariates of interest. For example, in a multi-site study comparing patients and healthy subjects, it is possible to correct distortions from site to site while conserving the health-related neuroanatomical variations, as described in J. P. Fortin et al. (2018). To account for the individual contribution of the different covariates, we applied several ComBat instances in a stepwise manner: first to remove sex-related effects, then age-related effects, and finally another instance of ComBat was applied to eliminate the scanner bias. To perform age correction, we treated age as a categorical variable taking the rounded integer value of the subject age.

### 2.5 Harmonization efficiency assessment

To evaluate the efficacy of the harmonization, we applied the nonparametric Kolmogorov-Smirnov two-sample test (K–S test; Darling, 1957; Massey, 1951; Smirnov, 1939) to the relative volumes of each ROI for each pair of scanners. The K–S test is a unidimensional test. Therefore, to verify the distinguishability of our multidimensional samples, the test needed to be performed for each pair of scanners on each of the 101 ROIs, as proposed in Garcia-Dias et al. (2019). Assuming that most of the systematic variation in the relative volume of the brain regions in healthy subjects can be explained by age, sex and the scanner bias, we expected that once we have eliminated differences associated with these variables, there should be substantial overlap among the relative volume distributions from different scanners. Therefore, if the harmonization is effective, the K–S test should fail to reject the null hypothesis. If the assumption that age, sex and scanner bias are the main sources of systematic bias is false, the K–S test should lead to the rejection of the null hypothesis for most pairs of scanners after harmonization. Since we are more concerned about type II errors, we did not perform any multiple comparison correction to the p-values.

### 2.6 Strength of the ComBat correction by ROI

The harmonization affects different regions with different magnitudes. To show how the ComBat harmonization affected each of the ROIs, we defined the correction ratio as the median volume of each region divided by the median correction provided by ComBat. For comparison, we used the coefficient of variation, $\text{CV}=100\;\times \frac{\sigma }{\mu }$, and the quartile-based coefficient of variation, $\text{QCV}=100\times \frac{Q_{3}-Q_{1}}{Q_{2}}.$

### 2.7 Strength of the ComBat correction for each covariate (sex, age, and scanner)

The ComBat harmonization process allows one to correct for one covariate while maintaining the variance from other covariates. In this way, we can measure the effect that each different covariate has on the final correction provided by ComBat. Since each scanner had different imbalances in terms of sex and age, we expected that each scanner would be corrected for each of the covariates to different degrees. As shown in Figs. 2 and 3, there was great variability in age and sex amongst scanners, with almost no overlap amongst some of the scanners. Therefore, to correct the scanner bias on the ROI relative volumes, we investigated how sex and age contribute to the differences among datasets. To this end, we measured the contribution of each covariate by taking the median of the absolute value of the ComBat corrections for all ROI volumes and summing all values per scanner. To make a reliable comparison among scanners, we divided the contribution of each covariate by the sum of all three contributions for this scanner, which we called ${\text{Δ}}_{tiv}$.

### 2.8 Neuroharmony training

We observed correlations between the relative volumes of ROIs with the IQMs of the images. Such observation is not unexpected since some of IQMs can directly influence the behavior of the preprocessing analysis. For example, this is clear for IQMs such as the FWHM (which measures the resolution of the image; see appendix A.1) that can affect the ability of FreeSurfer to distinguish the boundaries between regions. Similarly, some images with lower contrast-to-noise ratio could result in a systematic underestimation of the relative volume of a region due to the difficulties of distinguishing its boundaries. Here, we implemented random forest regressors (from the Scikit-learn^7^ python package, Buitinck et al., 2013; Pedregosa et al., 2011) to predict the harmonization corrections obtained with ComBat. We used the 68 IQMs generated by the MRIQC tool (listed in appendix A.1) as well as age, sex and the original relative volumes of the ROIs as input variables to predict the ComBat corrections for each ROI: RO${\text{I}}_{\text{correction}}$ ​= ​f$\left(\text{IQMs},\text{Age},\text{Sex},{\text{v}}_{\text{ROI}}\right)$ where, ${\text{v}}_{\text{ROI}}$ is the relative volume of the ROI. One model was trained per ROI. A comprehensive statistical description of each feature for each individual scanner can be found at garciadias.github.io/neuroharmony. In order to avoid the so-called “curse of dimensionality” and the inclusion of redundant variables, we performed a principal component analysis (PCA) (Wold et al., 1987) on the training dataset. This identified 20 principal components as the smallest number of principal components conserving 99% of the explained variance for all the input variables for all regions. In this way, we could generalize a rule that maps the IQMs to the corrections that ComBat would perform to the relative ROI volumes. This enabled us to estimate harmonization corrections for unseen scanners, as long as their image quality parameters fall within the range of parameters in our training sample.

We used a leave-one-scanner-out cross-validation strategy for hyperparameter search and selection for the random forest models. For the purpose of hyperparameter tuning only, we merged scanners with fewer than 30 images. This allowed us to greatly decrease the computational cost of the hyperparameter search and focus the training efforts on the scanners with a statistically representative sample. The merge of the datasets was applied only to the cross-validation split. The labels of the scanners were preserved during training and the final model was retrained without any scanners merged. During training, we also under-sampled the BIOBANK S01, as this would dominate the training sample for the model due to its very large size (n ​= ​9926). To this end, we randomly selected 555 subjects from BIOBANK S01, matching the number of subjects from TOMC-S02, the second-largest scanner sample. We also eliminated data from UCL S02, since ComBat failed to harmonize the data from this scanner (below).

**Fig. 4:**
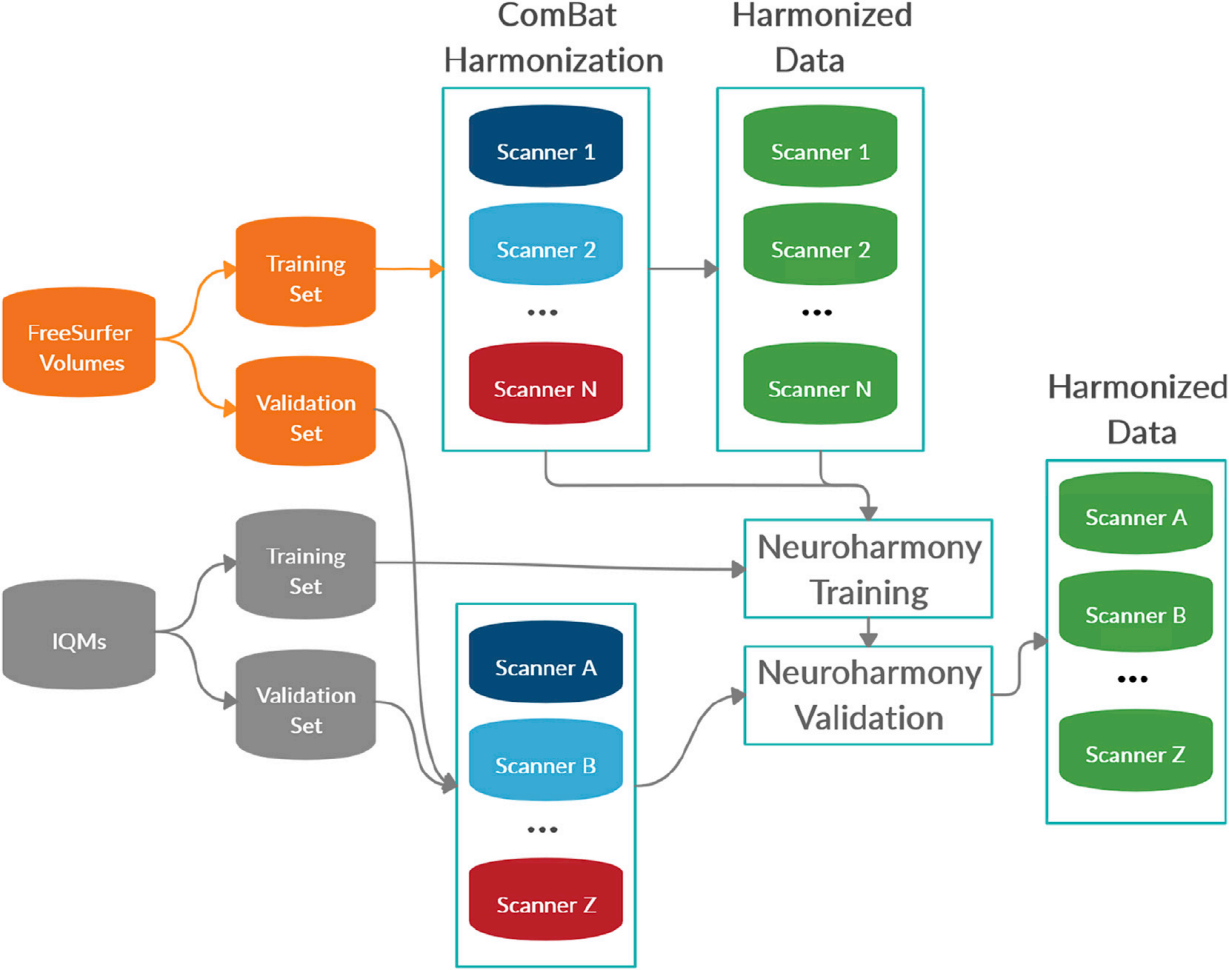
Diagram showing the data splits to train Neuroharmony.

For the validation of Neuroharmony we used 454 subjects from 16 scanners: ADHD200-NeuroIMAGE (n ​= ​22), ADHD200-Pittsburgh (n ​= ​20), BIOBANK-SCANNER02 (n ​= ​313), PPMI-SCANNER001 (n ​= ​5), PPMI-SCANNER012 (n ​= ​5), PPMI-SCANNER018 (n ​= ​5), PPMI-SCANNER032 (n ​= ​8), PPMI-SCANNER034 (n ​= ​15), PPMI-SCANNER057 (n ​= ​8), PPMI-SCANNER086 (n ​= ​5), PPMI-SCANNER088 (n ​= ​10), PPMI-SCANNER096 (n ​= ​11), PPMI-SCANNER120 (n ​= ​7), PPMI-SCANNER289 (n ​= ​6), PPMI-SCANNER290 (n ​= ​9), PPMI-SCANNER291 (n ​= ​5). To avoid any cross-contamination of the training and validation sets, we did not include these data in the ComBat harmonization or in the training of Neuroharmony. Fig. 4 illustrates the process splitting the scanner data to train Neuroharmony.

## 3 Results

### 3.1 Harmonization assessment

**Fig. 5:**
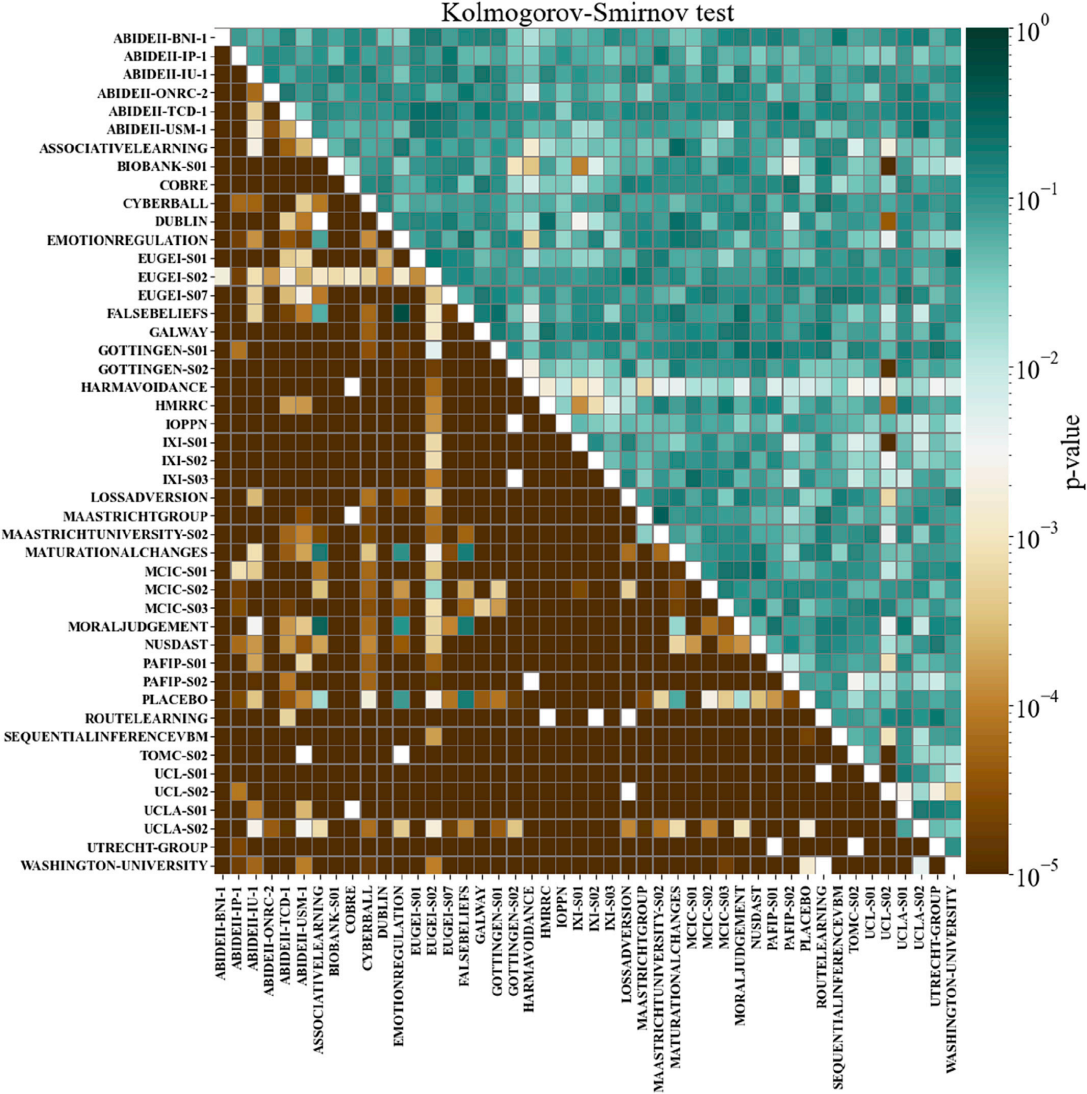
The minimum p-values for the K–S test among all ROIs, before and after ComBat harmonization. The cells under the main diagonal of the matrix represent the K–S test p-values before harmonization, while the values in the top of the main diagonal represent the p-values after the ComBat harmonization. Each cell corresponds to a pair of scanners. Cells are colored as shown on the color bar.

To evaluate the performance of ComBat harmonization, we ran the K–S test for every pair of scanners before and after harmonization, as shown in Fig. 5. The cells are colored according to the minimum p-value among all ROIs. This minimum p-value refers to the ROI with the worst harmonization correction among all ROIs for each pair of scanners. At a p-value of 0.001, most of the scanner pairs had distinguishable distributions of relative volumes before harmonization, but the harmonization was able to eliminate the bias between almost all pairs, raising the p-value above 0.001 for all ROIs. However, it is important to note that ComBat harmonization failed in some regions for some scanner pairs. For instance, the sample from the scanner UCL S02 remained distinguishable from the distribution of some scanners after harmonization. Investigation of the variables for which the harmonization failed revealed a noticeable double peak on the distributions, e.g. for the right and left cerebellum white matter.

### 3.2 Strength of the ComBat correction by ROI

**Fig. 6:**
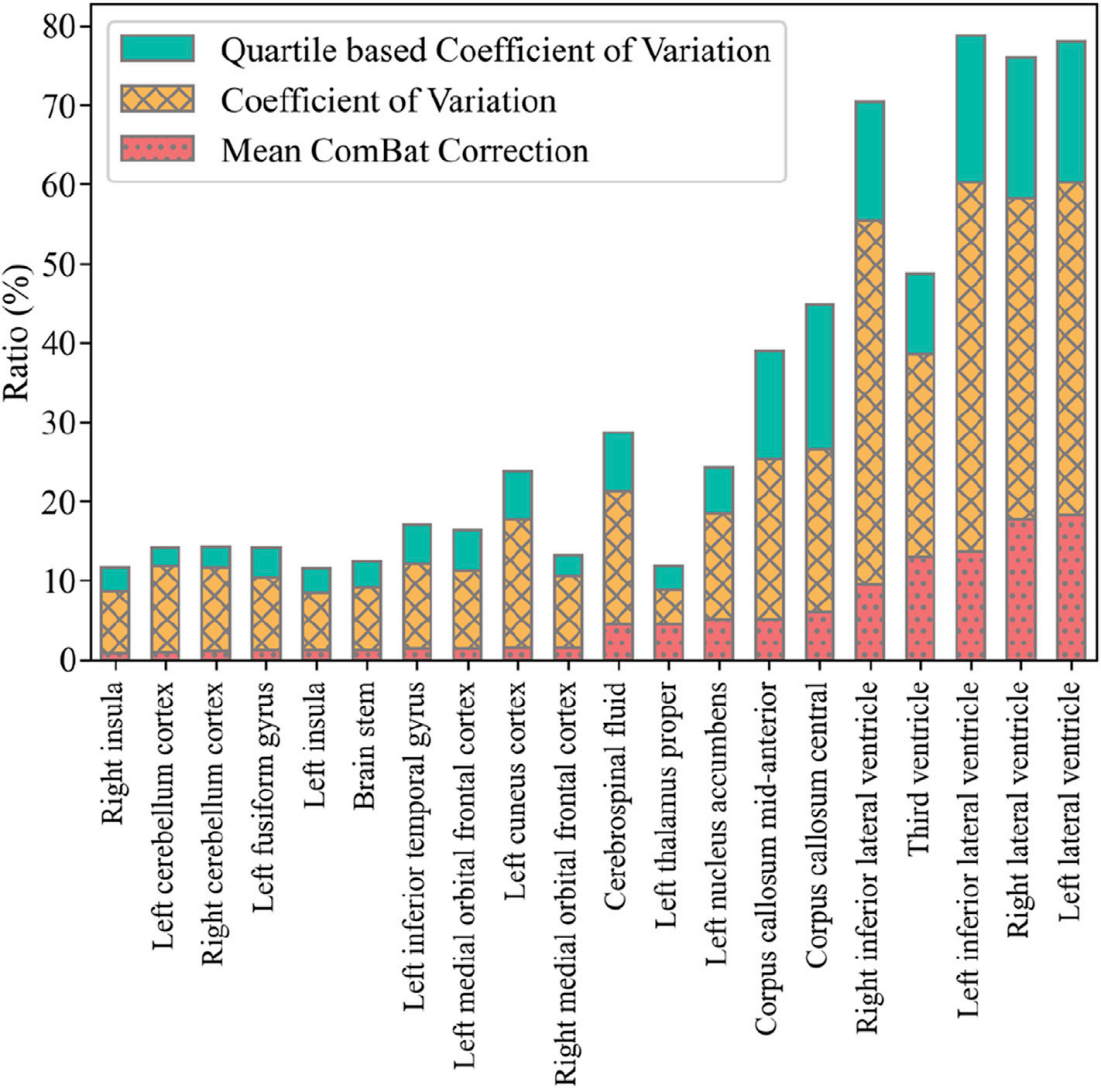
The median ROI volume divided by the median ComBat correction. From left to right, the first 10 ​bars show the ROIs with the smallest correction ratios while the next 10 show the ROIs with the largest correction ratio (red dotted bars). The X-hatched yellow bars show coefficient of variation and the green bars show the quartile-based coefficient of variation.

In Fig. 6, we showed only the 10 smallest correction ratios and the 10 largest correction ratios for clarity. We can see that the ventricles were especially affected by the harmonization, which means these regions were the ones with the largest divergent measurements among scanners. For example, the corrections account for more than 17% and 16% of the left and right lateral ventricle volumes, respectively. In our datasets, we observed that the lateral ventricles were also amongst the regions with the largest variability. Therefore, even when the corrections reached 17% of the mean volume of the region, the magnitude of the corrections was a fraction of the CV of the region. In other words, the scanner bias was small compared to the natural variability of the relative ROI volumes. In the supplementary material, we report a table showing how each of the ROIs was affected by the ComBat normalization together with their CV and QCV.

### 3.3 Strength of the ComBat correction for each covariate (sex, age, and scanner)

**Fig. 7:**
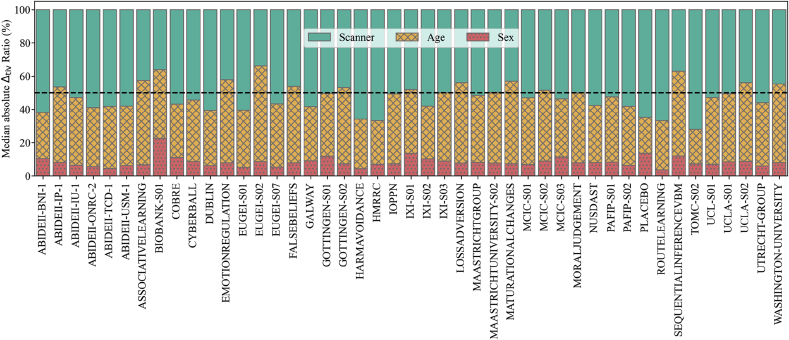
Relative contributions of each confound to the final ComBat correction. Each scanner is represented as a vertical bar divided in portions equivalent to the contributions that either scanner (green), age (yellow, X-hatched) or sex (red, filled with dots) made to the correction. The black dashed line marks the 50% level.

In Fig. 7 we show by what proportion each of the covariates affected the correction for each scanner. Correction for sex-related effects had a small impact, even on scanners dominated by one sex, as was the case of ABIDEII BNI 1. Age-related effects had a relatively higher contribution, but in most cases the dominant confound was the scanner of origin.

### 3.4 Validation

**Fig. 8:**
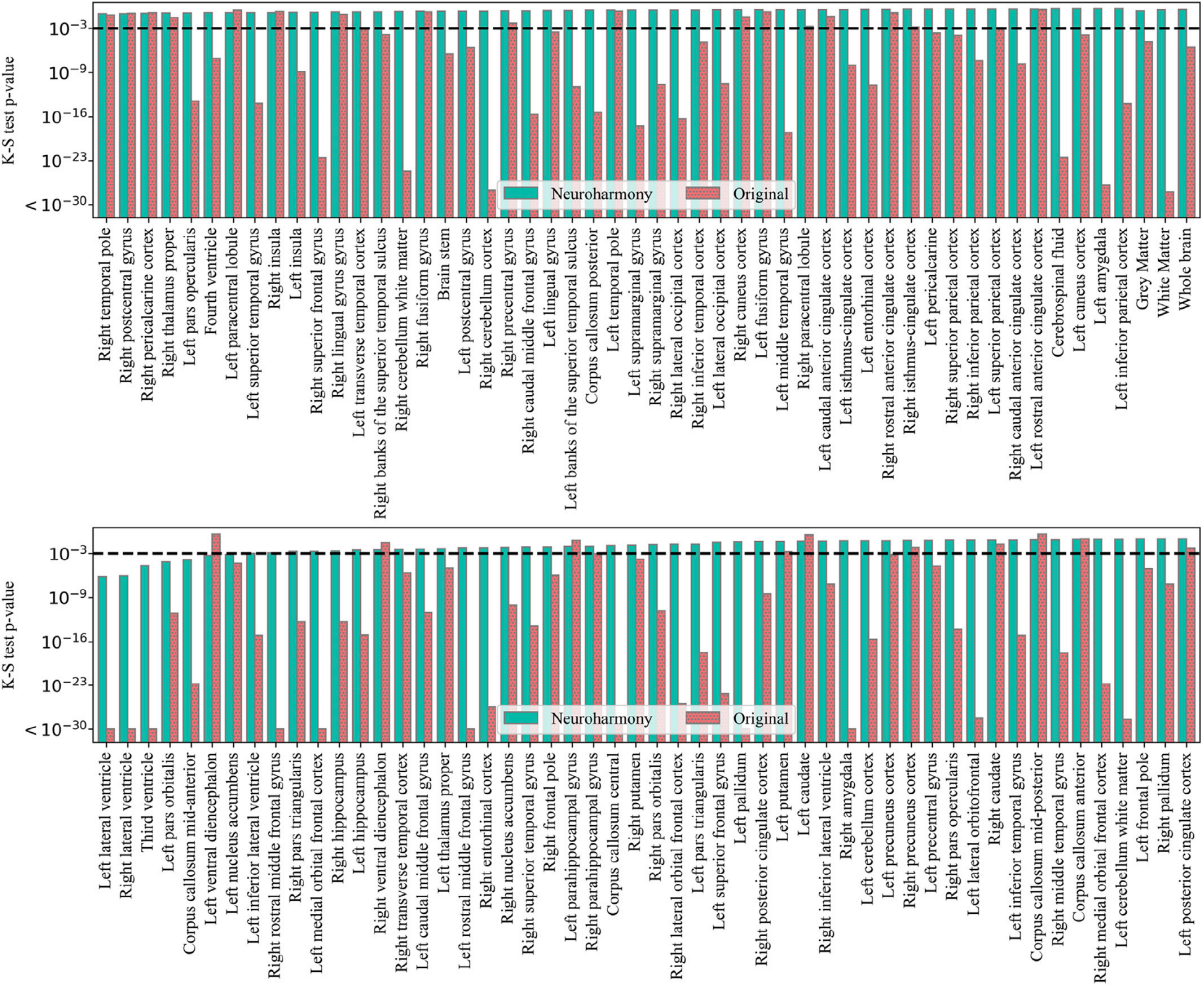
The p-values for the K–S test for the comparison between the validation set harmonized with Neuroharmony and the training sample harmonized with ComBat. From left to right, each bar in the pairs of bars represents the p-value of the K–S test for the data corrected by Neuroharmony (green) and the data without any correction (red, filled with dots). A horizontal black dashed line marks the 0.001 threshold.

Here, we present the results of the application of Neuroharmony to our external validation set. In Fig. 8, we show the p-values of the K–S tests comparing the validation set harmonized with Neuroharmony and the training set harmonized with ComBat. We see that Neuroharmony was able to achieve a p-value higher than 0.001 for almost all ROIs. Furthermore, Neuroharmony was also effective at the level of GM, WM and the whole brain with p-values of 0.455, 0.667 and 0.803, respectively. To calculate the effect of the harmonization at these levels, we added the values from all regions corresponding to GM or WM and compared these values before and after harmonization, as done for individual ROIs. It is important to remark that, as listed in the supplementary material, from the 101 ROIs only 7 corresponded to WM and 86 corresponded to GM, whilst 8 regions did not belong to either category. Therefore, a limitation of this approach was that these regions did not correspond to the whole brain, the totality of GM or WM, but it can illustrate how the tool would behave at these levels.

**Fig. 9:**
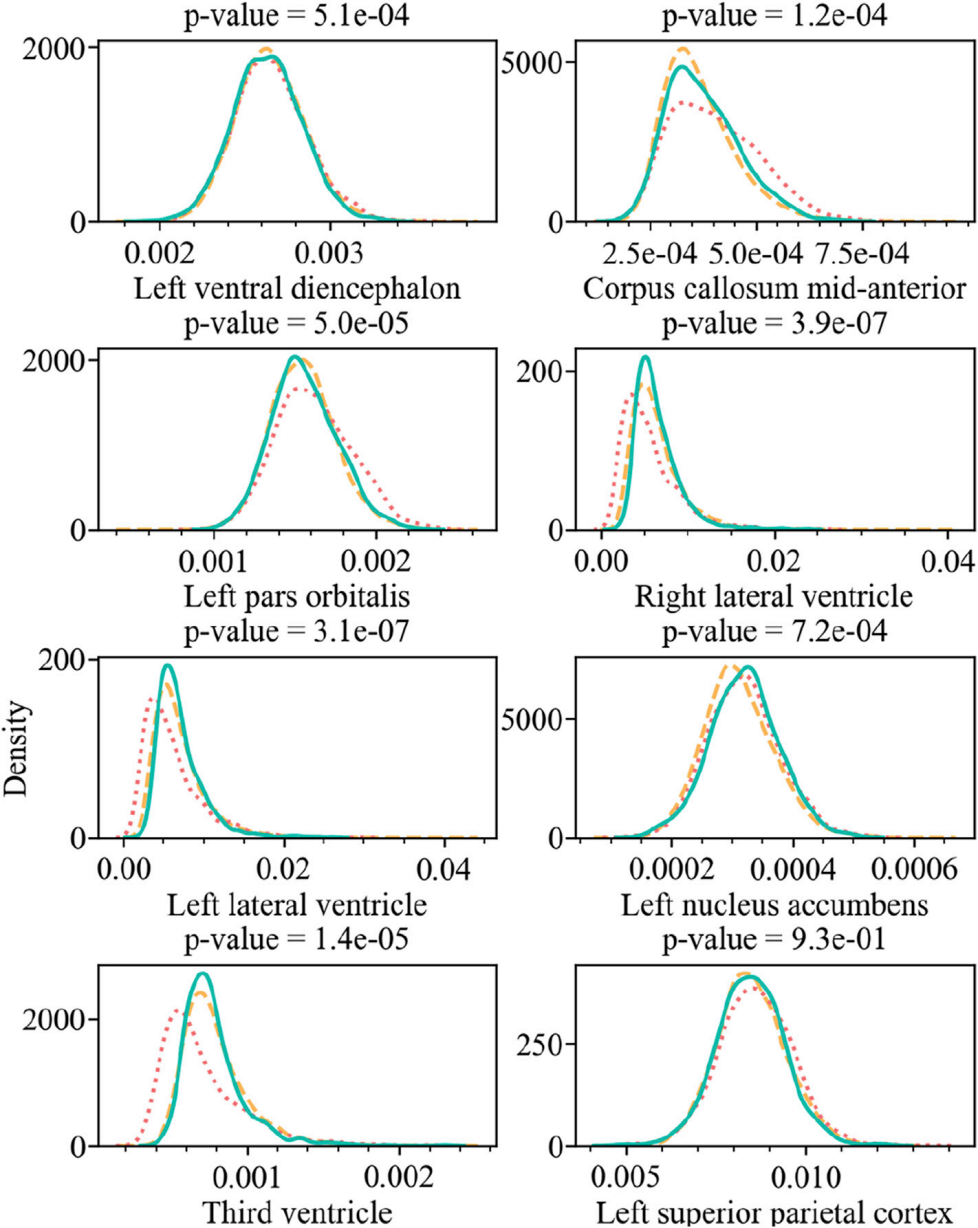
The kernel density plot for the relative volume of the regions as labelled in the x-axis of each plot. The title of each plot includes the p-value of the K–S test comparing the training set harmonized with ComBat (yellow dashed lines) and the validation set harmonized with Neuroharmony (green solid lines). The validation set without harmonization is shown as a red dotted line.

The only ROIs that were not completely corrected were the left ventral diencephalon, corpus callosum mid-anterior, left pars orbitalis, right lateral ventricle, left lateral ventricle, left nucleus accumbens, and third ventricle. However, in all cases except for the left ventral diencephalon, Neuroharmony was able to increase the p-value by orders of magnitude. In Fig. 9, we show the kernel density plot for the ComBat-harmonized training set, the ComBat-harmonized validation set, and the validation set *without harmonization for each of these* regions. We included the left superior parietal cortex, that achieved the 0.001 threshold, for comparison. The figure shows how the corrections were partially accomplished and that the harmonization approximated the density distributions relatively well.

## 4 Discussion

The aim of this study was to develop a new approach for harmonizing MRI data that would not require a statistically representative sample for each scanner and acquisition protocol, or a previous calibration of scanners. In essence, this involved training a machine learning tool, which we have called ‘Neuroharmony’, to capture the relationship between the intrinsic characteristics of the images and relative volume corrections for each ROI assigned by the ComBat harmonization.

Before training Neuroharmony on the ComBat outcomes, it was important to evaluate the behavior of the ComBat harmonization method, which we performed using a mega-dataset comprising of 15,026 healthy subjects from 62 scanners. This number of scanners exceeded the number of scanners of any previous application of ComBat in the literature. As expected, ComBat was capable of reducing scanner bias. Nevertheless, for some pairs of scanners, the null hypothesis of the K–S test was rejected, suggesting that between-scanner differences on certain brain regions remained after harmonization. This was likely caused by the presence of an unexpected double peak distribution in the relative volumes of these regions. ComBat performs multiplicative and additive corrections to the distributions, which are not able to eliminate this kind of distortion. The double peak observed in these regions was unexpected and it could not be explained by demographic imbalances. No differences in scanning protocol were reported. The source of this distortion needs further investigation. Furthermore, we found that different regions were affected by the ComBat harmonization to a different degree. We showed that the scanner-related corrections corresponded to a fraction of the natural variability of the relative volumes, indicating a high degree of neuroanatomical heterogeneity even amongst healthy subjects.

Having established that the ComBat harmonization tool behaved as expected, we proceeded to train a machine learning tool that used IQMs to predict the ComBat outcomes using the same mega-dataset. Consistent with our hypothesis, we found that it was possible to use the IQMs to predict the harmonization correction assigned by the ComBat tool. Overall, these results show that Neuroharmony can generalize the harmonization to unseen scanners. Neuroharmony was capable of providing corrections that eliminated clear differences between the data from the validation set and the rest of the data harmonized with ComBat. Improvements were observed even when the 0.001 threshold was not achieved.

To the best of our knowledge, Neuroharmony presents the first approach capable of providing harmonization for a single image of an unseen scanner. This approach has the potential to make a significant contribution towards bridging the gap between research – where the data have a known statistical distribution – and clinical applications of machine learning – where a single image may come from an unknown statistical distribution. In addition, this approach has the potential to reduce scanner bias in neuroimaging studies that aim to make single-subject inferences without necessarily using machine learning methods (C. Scarpazza, et al., 2013; C. Scarpazza et al., 2016).

The present study has a number of important limitations. Firstly, although our sample was very heterogeneous in terms of IQMs, we cannot guarantee that it covers all possible scanner configurations and acquisition protocols. For instance, if a scanner has a contrast-to-noise ratio outside the range of our training sample, we cannot guarantee an effective harmonization of the data. To mitigate this problem, the tool warns the user if any subjects fall outside the training range. Secondly, the model does not operate with the same accuracy for all regions. For example, mean absolute error was the lowest for the corpus callosum mid-posterior and anterior, and the highest for the lateral ventricles (both hemispheres), so the tool was more accurate in correcting the regions of the corpus collosum than the lateral ventricles. We suggest that the difficulties in correcting some of these regions might be explained by their high degree of variability. The ventricles, for example, were the regions with the largest CVs among all of the 101 ROIs. Such large variation is likely to be multifactorial, resulting from the contributions of variables such as handedness, craniotype, nutrition and health (Jacka et al., 2015; Luders et al., 2010; Zhuravlova et al., 2018). While sex, age and the IQMs were sufficient to eliminate systematic bias among scanners in the vast majority of regions, these additional sources of variability might explain the suboptimal performance of Neuroharmony in a subset of regions. An alternative explanation is that, given the nature of FreeSurfer segmentation, different regions might be affected by the quality of the image in different ways. A further explanation is that in some regions the relationships between the IQMs and the corrections established by ComBat are too complex to be generalized in our model. Further investigation is required to better understand the causes of the limited performance of Neuroharmony in these regions. Neuroharmony was developed to provide a solution for eliminating the bias in unseen scanners. However, when working with existing multisite datasets that include a statistically representative sample for each scanner, ComBat should be preferred. Here we demonstrate the efficacy of Neuroharmony on healthy subjects. At this stage we do not know whether the assumptions of the model hold when applied to patient data, and therefore we cannot conclude that the Neuroharmony tool is effective in reducing bias in the context of clinical studies. A further validation of the tool using patient data will be the focus of a future publication. It is important to note that we eliminated the variance due to age and sex to deal with the highly imbalanced nature of our sample; however, in some instances, it may be useful to preserve the variance from these covariates (e.g. in age prediction studies). Therefore, Neuroharmony allows the user to specify the variables for which variance should and should not be eliminated.

Despite these limitations, our initial validation suggests that our approach represents a significant step forward in the quest to develop clinically useful imaging-based tools. For example, Neuroharmony could be integrated within available clinical tools for single-subject inferences in brain disorders from MRI images. At present, none of these tools account for inter-scanner variability (see C. Scarpazza et al., 2020). Neuroharmony and the instructions on how to use it are available at https://github.com/garciadias/Neuroharmony.

## CRediT authorship contribution statement

**Rafael Garcia-Dias:** Conceptualization, Methodology, Software, Validation, Formal analysis, Investigation, Data curation, Writing - original draft, Visualization. **Cristina Scarpazza:** Data curation, Writing - review & editing. **Lea Baecker:** Data curation, Writing - review & editing. **Sandra Vieira:** Data curation, Writing - review & editing. **Walter H.L. Pinaya:** Data curation, Writing - review & editing, Funding acquisition. **Aiden Corvin:** Resources, Writing - review & editing. **Alberto Redolfi:** Resources, Writing - review & editing. **Barnaby Nelson:** Resources, Writing - review & editing. **Benedicto Crespo-Facorro:** Resources, Writing - review & editing. **Colm McDonald:** Resources, Writing - review & editing. **Diana Tordesillas-Gutiérrez:** Resources, Writing - review & editing. **Dara Cannon:** Resources, Writing - review & editing. **David Mothersill:** Resources, Writing - review & editing. **Dennis Hernaus:** Resources, Writing - review & editing. **Derek Morris:** Resources, Writing - review & editing. **Esther Setien-Suero:** Resources, Writing - review & editing. **Gary Donohoe:** Resources, Writing - review & editing. **Giovanni Frisoni:** Resources, Writing - review & editing. **Giulia Tronchin:** Resources, Writing - review & editing. **João Sato:** Resources, Funding acquisition, Writing - review & editing. **Machteld Marcelis:** Resources, Writing - review & editing. **Matthew Kempton:** Resources, Writing - review & editing. **Neeltje E.M. van Haren:** Resources, Writing - review & editing. **Oliver Gruber:** Resources, Writing - review & editing. **Patrick McGorry:** Resources, Writing - review & editing. **Paul Amminger:** Resources, Writing - review & editing. **Philip McGuire:** Resources, Writing - review & editing. **Qiyong Gong:** Resources, Writing - review & editing. **René S. Kahn:** Resources, Writing - review & editing. **Rosa Ayesa-Arriola:** Resources, Writing - review & editing. **Therese van Amelsvoort:** Resources, Writing - review & editing. **Victor Ortiz-García de la Foz:** Resources, Writing - review & editing. **Vince Calhoun:** Resources, Funding acquisition, Writing - review & editing. **Wiepke Cahn:** Resources, Writing - review & editing. **Andrea Mechelli:** Conceptualization, Writing - review & editing, Supervision, Project administration, Funding acquisition.

## Figures and Tables

**Fig. 10:**
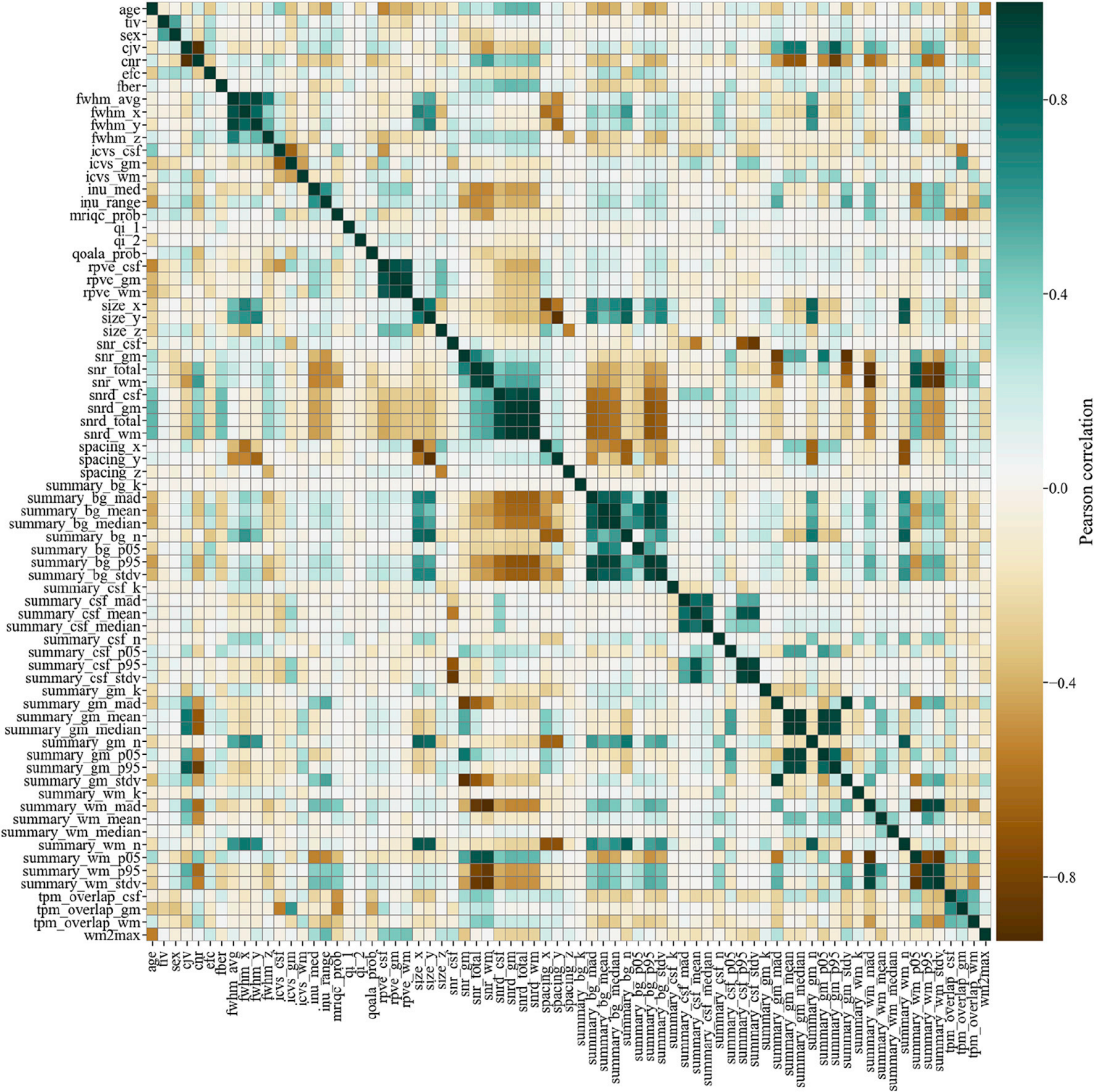
The Pearson correlation matrix for all IQMs, sex and age.

## Appendix A.1 Image quality metrics

Let $x_{j}$ be the intensity of each voxel (X ​= ​{$x_{1},x_{2},\ldots ,x_{j},\ldots ,x_{N}$}) in an image with N voxels. Therefore, the mean brightness of the image is defined as $\mu =\frac{\sum_{j=1}^{N}x_{j}}{N}$ and the standard deviation as $\sigma =\frac{1}{N}\sum_{j=1}^{N}{\left(x_{j}-\mu \right)}^{2}$. When these quantities are measured considering only the pixels of a given tissue, we use sub-indexes WM for white matter and GM for grey matter.

• CJV: Coefficient of joint variation between white matter and grey matter. The CJV is defined as:

$$\text{CJV}=\text{ ​}\frac{\sigma_{WM}^{\text{ ​}}+\sigma_{GM}^{\text{ ​}}}{\mu_{WM}^{\text{ ​}}-\mu_{WM}^{\text{ ​}}}.$$

• CNR: The contrast-to-noise rate evaluates how the contrast between grey and white matter relates to the noise on the image. The CNR is defined as:

$$CNR=\text{ ​}\frac{\mu_{GM}^{\text{ ​}}-\mu_{WM}^{\text{ ​}}}{\sigma_{background}^{\text{ ​}}}.$$

• SNR: The signal-to-noise ratio evaluates how the mean signal measurements relate to the noise in the image. The SNR is measured separately for GM, WM, CSF and for the whole brain. The SNR is defined as:

$$SNR_{tissue}=\frac{\mu_{tissue}^{\text{ ​}}}{\sigma_{tissue}^{\text{ ​}}}$$

• SNRD: The Dietrich signal-to-noise ratio compares the mean sign on the brain tissue with the standard deviation in the background with a correction factor. The SNRD can be written as

$$SNRD=\frac{\mu_{brain}}{\sqrt{\frac{2}{4-\pi }}\;\sigma_{background}}.$$

• INU: The intensity non-uniformity index measures inhomogeneities in the brightness of the image (Tustison et al., 2010).
• QI_1_: The ratio of ‘bad’ voxels on the background of the image. This is measured based on a statistical approach. The voxels in the background that are out of a range covering 10 median absolute deviations around the median value are considered to be artifacts. The bounders of the image are discarded in this process. The method is described by Mortamet et al. (2009).
• QI_2_: A measurement of the goodness of fitting of the background distributions with the expected probability density function, excluding the artifact voxels accounted by QI_1_. The method is described by Mortamet et al. (2009).
• EFC: The entropy focus criterion is a measure of the Shannon entropy that indicates the presence of blurring and ghosting. This quantity is an estimation of how well the brightness throughout the image is distributed. The method is described by Atkinson et al. (1997). The energy E is calculated as

$$E=-\sum_{\text{j}=1}^{\text{N}}\frac{{\text{x}}_{\text{j}}}{{\text{B}}_{max}}\ln\left[\frac{{\text{x}}_{\text{j}}}{{\text{B}}_{max}}\right].$$

where $B_{max}$ is the maximum entropy, $B_{max}=\sqrt{\sum_{j=1}^{N}x_{j}^{2}}$. With this, the EFC can be written as

$$EFC=\left(\frac{N}{\sqrt{N}}\;\log\sqrt{N^{-1}}\right)E.$$

• FBER: The foreground-background energy ratio. Similar to SNR, this is an estimation of how much brighter the image of the brain is in relation to the background.

$$FBER=\frac{E_{brain}}{E_{background}}.$$

• WM2MAX: This index measures how close to the maximum count the mean brightness of the white matter region is. The mean white matter value is compared to the 99.95 percentile ($P_{99.95}\left(X\right)$). Ideally, this value is in the range of 0.6–0.8. The WM2AX is defined by

$$WM2MAX=\frac{\mu_{WM}}{P_{99.95}\left(X\right)}.$$

• FWHM: The Full width at half maximum is a measure of the resolution of the image. Lower values indicate a higher resolution, while higher values indicate a blurrier image. The FWHM is measured in a two-dimensional plane. Therefore, four values of FWHM are presented, x, y, z and the average of the three. X, y and z correspond to the coronal, transverse and sagittal planes. The FWHM is defined as

$$FWHM_{m}=\sqrt{-{\left[4\;\ln\left\{\left(1-\frac{\sigma_{X_{i+1,j}^{m}-X_{i,j}^{m}}^{2}}{2\sigma_{X_{i,j}^{m}}^{2}}\right\}\right)\right]}^{-1}},m\in \left\{x,y,z\right\}.$$

• ICVs: The intracranial volume of each of each tissue (CSF, GM, and WM). Deviations from the normal expected values can indicate poor image quality.
• rPVE: The residual partial volume effect accounts for the potential errors generated by counting the volumes in voxels on the interface of different tissues. The rPVE is calculated for each of the brain tissues (CSF, GM, and WM).
• Summary statistics: A collection of statistical metrics that summarize the distribution of voxel brightness in the different regions (background, CSF, WM, and GM). The measured quantities include mean, standard deviation, percentiles 5% and 95%, and kurtosis.
• TPMs: This metric of tissue probability maps establishes a comparison between the different tissues with the templates from Fonov et al. (2009). The index measures the overlap between the different tissues (CSF, GM, and WM) in the template with those in the subject.

On Fig. 10 we show the Pearson correlation among all regression features.

## Footnotes

1 https://mriqc.readthedocs.io.

2 Structural MRI data were obtained from www.nitrc.org.

3 The imaging data and demographic information was collected and shared by [University of Iowa, University of Minnesota, University of New Mexico, Massachusetts General Hospital] the Mind Research Network supported by the Department of Energy under Award Number DE-FG02-08ER64581.

4 Data used in the preparation of this article were obtained from the Parkinson’s Progression Markers Initiative (PPMI) database (www.ppmi-info.org/data). For up-to-date information on the study, visit www.ppmi-info.org.

5 https://doi.org/10.1016/j.neuroimage.2020.117127

6 https://surfer.nmr.mgh.harvard.edu.

7 https://scikit-learn.org/stable/index.html.
