# Supplementary material for: Neuroharmony: A new tool for harmonizing volumetric MRI data from unseen scanners
Source: Neuroimage. 2020 Oct 15;220:117127. doi: 10.1016/j.neuroimage.2020.117127 (PMC7573655; doi:10.1016/j.neuroimage.2020.117127)
Supplement: Multimedia component 1 [file mmc1.docx]

Table 1: Scanner acquisition parameters, availability and number of subjects.

|  | N subjects | Source | Manufacturer | Field Intensity (T) | Sequence | Plan of acquisition | TE (ms) | TR (ms) | Flipangle (°) | Voxel size (mm) | Matrix dimension |
| --- | --- | --- | --- | --- | --- | --- | --- | --- | --- | --- | --- |
| ABIDEII BNI 1 | 29 | Freely available | PhilipsIngenia | 3 | MPRAGE | Axial | 3.1 | 6.7 | 9 | 1.1 x 1.1 x 1.2 | 244 x 227x 170 |
| ABIDEII IP 1 | 23 | Freely available | PhilipsAchieva | 1.5 | MPRAGE | Axial | 5.6 | 25 | 30 | 1.0 x 1.0 x 1.0 | 240 x 240 x 170 |
| ABIDEII IU 1 | 20 | Freely available | SiemensTriTim | 3 | TFL | Axial | 2.3 | 2400 | 8 | 0.7 x 0.7 x 0.7 | 320 x 320 x 256 |
| ABIDEII ONRC 2 | 29 | Freely available | SIEMENS MAGNETOM Skyrasyngo | 3 | MPRAGE | Axial | 2.88 | 2200 | 13 | 0.8 x 0.8 x 0.8 | 220 x 320 x 208 |
| ABIDEII TCD 1 | 7 | Free available | PhilipsAchieva | 3 | MPRAGE | Axial | 3.9 | 8.4 | 8 | 0.9 x 0.9 x 0.9 | 256 x 256 x 190 |
| ABIDEII USM 1 | 13 | Freely available | SiemensTriTim | 3 | MPRAGE | Axial | 2.91 | 900 | 9 | 1.0 x 1.0 x 1.2 | 256 x 256 x 160 |
| ADHD200 NEUROIMAGE | 22 | Freely available | Siemens Magnetom Avanto | 1.5 | - | Coronal | 2.95 | 2730 | 7 | 1.0 x 1.0 x 1.0 | 256 x 256 x 176 |
| ADHD200 PITTSBURGH | 20 | Freely available | Siemens Magnetom Trio Tim Syngo | 3 | MPRAGE | Sagittal | 3.43 | 2100 | 8 | 1.0 x 1.0 x 1.0 | 256 x 208 x 176 |
| ASSOCIATIVE LEARNING | 100 | Freely available | SiemensVerio | 3 | MPRAGE | Sagittal | 409 | 3200 | 9 | 1.0 x 1.0 x 1.0 | 176 x 256 x 256 |
| BIOBANK S01 | 9966 | Private dataset | SiemensSkyra | 3 | MPRAGE | Sagittal | 2.01 | 2000 | 8 | 1.0 x 1.0 x 1.0 | 208 x 256 x 256 |
| BIOBANK S02 | 313 | Private dataset | SiemensSkyra | 3 | MPRAGE | Sagittal | 2.01 | 2000 | 8 | 1.0 x 1.0 x 1.0 | 208 x 256 x 256 |
| COBRE | 84 | Freely available | Siemens | 3 | MPRAGE | Sagittal | 3.5 | 1.65 | 7 | 1.0 x 1.0 x 1.0 | 256 x 256 x 176 |
| CYBERBALL | 16 | Freely available | Siemens Magnetom Verio | 3 | MPRAGE | - | 2.98 | 2300 | 90 | 1.0 x 1.0 x 1.0 | 256 x 256 x 160 |
| DUBLIN | 146 | Private Dataset | Philips Intera Achieva | 3 | TFE | - | 3.8 | 8.4 | 8 | 0.9 x 0.9 x 0.9 | 256 x 256 x 180 |
| EMOTION REGULATION | 118 | Freely available | GE SIGNAHDx | 1.5 | - | - | 40 | 2000 | 60 | 0.85 x 0.85 x 1.5 | 256 x 256 x 116 |
| EU GEI S01 | 15 | Private Dataset | GE Discovery | 3 | MPRAGE | Sagittal | 3.03 | 7.34 | 11 | 1.0 x 1.0 x 1.2 | 256 x 256 x 166 |
| EU GEI S02 | 5 | Private Dataset | Philips Healthcare Ingenia | 3 | MPRAGE | Sagittal | 3.15 | 6.81 | 9 | 1.0 x 1.0 x 1.2 | 256 x 256 x 170 |
| EU GEI S07 | 17 | Private Dataset | Siemens TIM Trio | 3 | MPRAGE | Sagittal | 2.49 | 2300 | 9 | 1.0 x 1.0 x 1.2 | 256 x 240 x 176 |
| FALSEBELIEFS | 135 | Freely available | Siemens | 3 | - | - | 35 | 2000 | - | 1.2 x 1.2 x 1.2 | 144 x 192 x 192 |
| GALWAY | 54 | Private Dataset | Siemens Magnetom scanner | 1.5 | MPRAGE | Sagittal | 4.38 | 1140 | 15 | 0.45 x 0.45 x 0.9 | 256 x 256 x 160 |
| GOTTINGEN S01 | 58 | Private Dataset | Siemens Sonata | 1.5 | MPRAGE | Sagittal | 3.26 | 1900 | 15 | 1.0 x 1.0 x 1.0 | 256 x 256 x 176 |
| GOTTINGEN S02 | 323 | Private Dataset | Siemens Tim Trio | 3 | MPRAGE | Sagittal | 3.42 | 2250 | 9 | 1.0 x 1.0 x 1.0 | 256 x 256 x 176 |
| HARMAVOIDANCE | 85 | Freely available | AchievaPhilips, Best | 3 | TFE | Axial | 3.7 | 7.6 | 8 | 1 x 1 x 2 | 240 x 240 x 200 |
| HMRRC | 219 | Private Dataset | EXCITE GE | 3 | MPRAGE | Axial | 3.4 | 8.5 | 12 | 1.0 x 0.47 x 0.47 | 256 x 256 x 156 |
| IOPPN | 22 | Private Dataset | GE Medical Systems | 3 | FSPGR | Coronal | 7.08 | 28.32 | 20 | 1.09 x 1.09 x 1.1 | 256 x 256 x 196 |
| IXI S01 | 367 | Free available | PhilipsIntera | 3 | - | - | 4.6 | 9.6 | 8 | 1 x 1 x 1.2 | 256 x 256 x 130 |
| IXI S02 | 209 | Free available | PhilipsGyroscanIntera | 1.5 | - | - | 4.6 | 9.8 | 8 | 1 x 1 x 1.2 | 256 x 256 x 150 |
| IXI S03 | 39 | Free available | GE | 1.5 | - | - | 2.4 | 5.8 | 20 | 1 x 1 x 1.2 | 256 x 256 x 146 |
| LOSS AVERSION | 103 | Free available | Siemens Skyra | 3 | - | - | - | 2.4 | 8 | 0.8 x 0.8 x 0.8 | 256 x 256 x 256 |
| MAASTRICHT UNIVERSITY S02 | 88 | Private Dataset | Philips Intera | 3 | - | Axial | 4.6 | 9.8 | 8 | 1.17 x 1.17 x 1.2 | 192 x 152 x 120 |
| MAASTRICHT GROUP | 67 | Private dataset | Siemens Magnetom Allegra | 3 | MPRAGE | - | - | 2250 | 9 | 1 x 1 x 1 | 256 x 256 x 192 |
| MATURATIONAL CHANGES | 74 | Freely available | MAGNETOM Allegra | 3 | MPRAGE | Sagittal | 3.04 | 1570 | 8 | 0.78 x 0.78 x 1 | 192 x 256 x 256 |
| MCIC S01 | 42 | Free available | Siemens Sonata | 1.5 | - | Coronal | 4.76 | 12 | 20 | 0.625 x 0.625 x 1.5 | 256 x 256 x 128 |
| MCIC S02 | 19 | Free available | Siemens Trio | 3 | - | Coronal | 3.79 | 2530 | 7 | 0.625 x 0.625 x 1.5 | 256 x 256 x 128 |
| MCIC S03 | 20 | Free available | Siemens | 1.5 | - | Coronal | 4.76 | 12 | 20 | 0.625 x 0.625 x 1.5 | 256 x 256 x 128 |
| MORAL JUDGEMENT | 109 | Freely available | Siemens Trio Tim | 3 | MPRAGE | Axial | 3.39 | 2000 | 9 | 3 x 3 x 4 | 128 x 256 x 256 |
| NUSDAST | 34 | Freely available | Siemens Vision | 1.5 | Turbo-FLASH, MPRAGE | - | 5.4 | 20 | 30, 10 | 1 x 1 x 1 | 256 x 256 x 180 |
| PAFIP-S01 | 106 | Private dataset | Philips Achieva | 3 | - | Sagittal | 3.7 | 8.2 | 8 | 0.94 x 0.94 x 1 | 256 x 256 x 160 |
| PAFIP-S02 | 71 | Private dataset | Signa GE | 1.5 | SPGR | Coronal | 5 | 24 | 45 | 1.02 x 1.02 x 1.5 | 256 x 256 x 124 |
| PLACEBO | 43 | Freely available | Siemens Trio | 3 | MPRAGE | - | 3.36 | 2500 | 9 | 1 x 1 x 1 | 256 x 256 x 160 |
| PPMI S001 | 5 | Freely available | - | - | - | - | - | - | - | 1.2 x 1 x 1 | 152 x 256 x 256 |
| PPMI S012 | 5 | Freely available | Siemens Trio Tim | 3 | MPRAGE GRAPPA | Sagittal | 2.98 | 2300 | 9 | 1 x 1 x 1 | 241 x 256 x 176 |
| PPMI S018 | 5 | Freely available | - | - | - | Axial | - | - | - | 0.5 x 0.5 x 1 | 512 x 512 x 156 |
| PPMI S032 | 8 | Freely available | Siemens Trio Tim | 3 | MPRAGE, MPRAGE GRAPPA | Sagittal | 2.75 | 2300 | 10 | 1 x 1 x 1 | 256 x 256 x 176 |
| PPMI S034 | 15 | Freely available | Siemens Trio Tim | 3 | MPRAGE | Sagittal | 3.93 | 2300 | 9 | 1 x 1 x 1 | 240 x 256 x 176 |
| PPMI S057 | 8 | Freely available | Philips Medical Systems | 3 | MPRAGE | Sagittal | 3.2 | 2300 | 8 | 1 x 1 x 1 | 288 x 288 x 170 |
| PPMI S086 | 5 | Freely available | Siemens | 3 | MPRAGE GRAPPA | Sagittal | 2.98 | 2300 | 9 | 1 x 1 x 1 | 240 x 256 x 176 |
| PPMI S088 | 10 | Freely available | Siemens Trio Tim | 3 | MPRAGE GRAPPA | Sagittal | 2.98 | 2300 | 9 | 1 x 1 x 1 | 240 x 256 x 176 |
| PPMI S096 | 11 | Freely available | Siemens Trio Tim | 3 | MPRAGE | Sagittal | 2.27 | 1900 | 9 | 1 x 1 x 1 | 256 x 256 x 176 |
| PPMI S120 | 7 | Freely available | Siemens Trio Tim | 3 | MPRAGE GRAPPA | Sagittal | 2.98 | 2300 | 9 | 1 x 1 x 1 | 240 x 256 x 176 |
| PPMI S289 | 6 | Freely available | Siemens Trio Tim | 3 | MPRAGE GRAPPA | Sagittal | 2.98 | 2300 | 9 | 1 x 1 x 1 | 240 x 256 x 176 |
| PPMI S290 | 9 | Freely available | Siemens Trio Tim | 3 | MPRAGE GRAPPA | Sagittal | 2.98 | 2300 | 9 | 1 x 1 x 1 | 240 x 256 x 176 |
| PPMI S291 | 5 | Freely available | Siemens Verio | 3 | MPRAGE GRAPPA | Sagittal | 2.98 | 2300 | 9 | 1 x 1 x 1 | 240 x 256 x 176 |
| ROUTE LEARNING | 40 | Freely available | Siemens Allegra | 3 | - | Sagittal | 3.93 | 2500 | 8 | 1 x 1 x 1 | 176x 256 × 256 |
| SEQUENTIAL INFERENCE VBM | 74 | Freely available | Siemens TrioMagnetom | 3 | MPRAGE | Sagittal | 2.34 | 1550 | 9 | 1 x 1 x 1 | 256 x 256 x 176 |
| TOMC-S02 | 555 | Private Dataset | Gyroscan_NT | 1 | TFE | Sagittal | 5 | 20 | 30 | 0.85 x 0.85 x 1.6 | 256 x 256 x 170 |
| UCL S01 | 192 | Private Dataset | Siemns Avanto | 1.5 | FLASH | Axial | 5.6 | 12 | 19 | 1 x 1 x 1 | 256 x 240 x 160 |
| UCL S02 | 476 | Private Dataset | Siemns Avanto | 1.5 | MPRAGE | Axial | 3.57 | 2730 | 7 | 1 x 1 x 1 | 256 x 240 x 176 |
| UCLA S01 | 89 | Freely available | Siemens TriosyngoMR B15 | 3 | MPRAGE | Sagittal | 2.26 | 1900 | - | 1 x 1 x 1 | 176 x 256 x 256 |
| UCLA S02 | 21 | Freely available | Siemens TriosyngoMR B17 | 3 | MPRAGE | Sagittal | 2.26 | 1900 | - | 1 x 1 x 1 | 176 x 256 x 256 |
| UTRECHT GROUP | 130 | Private dataset | PhilipsAchieva | 1.5 | 3D-FFE | Coronal | 4.6 | 30 | 30 | 1 x 1 x 1.2 | 256 x 256 x 170 |
| WASHINGTON UNIVERSITY | 120 | Freely available | Siemens MAGNETOM Trio | 3 | - | - | 30 | 2400 | 8 | 1 x 1 x 1 | 256 x 256 x 256 |

Table 2: List of the region of interest (ROIs) along with their ComBat correction

ratio, coefficient of variability (CV) and quartile based coefficient of variation.

| **Region of Interest** | **Correction ratio (%)** | **CV (%)** | **QCV (%)** |
| --- | --- | --- | --- |
| Brain stem | 1.20 | 9.10 | 12.30 |
| Cerebrospinal fluid | 4.40 | 21.30 | 28.70 |
| Corpus callosum anterior | 2.90 | 15.20 | 20.50 |
| Corpus callosum central | 6.00 | 26.60 | 44.80 |
| Corpus callosum mid-anterior | 5.10 | 25.30 | 38.90 |
| Corpus callosum mid-posterior | 4.00 | 18.60 | 23.70 |
| Corpus callosum posterior | 4.00 | 15.60 | 20.50 |
| Fourth ventricle | 2.90 | 32.80 | 39.40 |
| Left amygdala | 3.00 | 12.10 | 15.10 |
| Left banks of the superior temporal sulcus | 3.30 | 17.20 | 21.60 |
| Left caudal anterior cingulate cortex | 2.50 | 24.20 | 34.00 |
| Left caudal middle frontal gyrus | 2.50 | 15.90 | 22.00 |
| Left caudate | 1.90 | 11.20 | 14.90 |
| Left cerebellum cortex | 0.90 | 11.80 | 14.10 |
| Left cerebellum white matter | 2.90 | 28.70 | 29.90 |
| Left cuneus cortex | 1.40 | 17.60 | 23.80 |
| Left entorhinal cortex | 2.40 | 18.70 | 24.10 |
| Left frontal pole | 2.70 | 17.00 | 21.50 |
| Left fusiform gyrus | 1.10 | 10.40 | 14.20 |
| Left hippocampus | 1.90 | 9.20 | 12.00 |
| Left inferior lateral ventricle | 13.60 | 60.30 | 78.80 |
| Left inferior parietal cortex | 2.90 | 13.70 | 18.90 |
| Left inferior temporal gyrus | 1.40 | 12.10 | 17.00 |
| Left insula | 1.20 | 8.40 | 11.50 |
| Left isthmus-cingulate cortex | 2.60 | 13.40 | 19.20 |
| Left lateral occipital cortex | 1.70 | 12.70 | 16.70 |
| Left lateral orbitofrontal | 1.90 | 10.20 | 14.10 |
| Left lateral ventricle | 18.40 | 60.30 | 78.00 |
| Left lingual gyrus | 1.80 | 14.80 | 20.40 |
| Left medial orbital frontal cortex | 1.40 | 11.20 | 16.40 |
| Left middle temporal gyrus | 2.50 | 11.90 | 17.00 |
| Left nucleus accumbens | 5.10 | 18.50 | 24.30 |
| Left pallidum | 1.50 | 11.50 | 15.40 |
| Left paracentral lobule | 2.40 | 12.40 | 16.90 |
| Left parahippocampal gyrus | 2.80 | 14.40 | 18.50 |
| Left pars opercularis | 3.50 | 16.70 | 21.70 |
| Left pars orbitalis | 2.90 | 14.40 | 20.30 |
| Left pars triangularis | 4.00 | 16.70 | 23.60 |
| Left pericalcarine | 1.60 | 21.60 | 29.50 |
| Left postcentral gyrus | 1.80 | 11.30 | 14.30 |
| Left posterior cingulate cortex | 1.70 | 13.70 | 17.50 |
| Left precentral gyrus | 1.80 | 10.30 | 14.00 |
| Left precuneus cortex | 1.50 | 10.30 | 13.90 |
| Left putamen | 2.20 | 10.30 | 13.60 |
| Left rostral anterior cingulate cortex | 2.00 | 16.80 | 22.60 |
| Left rostral middle frontal gyrus | 4.20 | 13.80 | 19.70 |
| Left superior frontal gyrus | 2.50 | 10.60 | 15.50 |
| Left superior parietal cortex | 2.20 | 11.90 | 16.00 |
| Left superior temporal gyrus | 2.20 | 11.30 | 15.60 |
| Left supramarginal gyrus | 2.10 | 13.60 | 17.80 |
| Left temporal pole | 2.10 | 15.00 | 19.50 |
| Left thalamus proper | 4.40 | 8.80 | 11.80 |
| Left transverse temporal cortex | 2.00 | 17.20 | 23.90 |
| Left ventral diencephalon | 1.50 | 8.00 | 10.80 |
| Right amygdala | 2.00 | 10.90 | 14.10 |
| Right banks of the superior temporal sulcus | 2.40 | 14.30 | 19.00 |
| Right caudal anterior cingulate cortex | 2.30 | 22.40 | 29.20 |
| Right caudal middle frontal gyrus | 2.80 | 17.00 | 22.10 |
| Right caudate | 1.70 | 11.00 | 14.00 |
| Right cerebellum cortex | 1.10 | 11.60 | 14.20 |
| Right cerebellum white matter | 2.80 | 28.20 | 30.60 |
| Right cuneus cortex | 1.90 | 16.40 | 21.20 |
| Right entorhinal cortex | 3.00 | 19.20 | 24.20 |
| Right frontal pole | 2.50 | 16.80 | 21.80 |
| Right fusiform gyrus | 1.60 | 10.80 | 15.00 |
| Right hippocampus | 2.10 | 9.00 | 11.70 |
| Right inferior lateral ventricle | 9.50 | 55.50 | 70.30 |
| Right inferior parietal cortex | 2.20 | 12.10 | 16.90 |
| Right inferior temporal cortex | 1.40 | 11.40 | 15.30 |
| Right insula | 0.80 | 8.60 | 11.60 |
| Right isthmus-cingulate cortex | 1.50 | 13.10 | 17.60 |
| Right lateral occipital cortex | 2.00 | 13.20 | 17.00 |
| Right lateral orbital frontal cortex | 1.60 | 10.80 | 15.30 |
| Right lateral ventricle | 17.70 | 58.30 | 76.00 |
| Right lingual gyrus gyrus | 1.90 | 13.80 | 17.20 |
| Right medial orbital frontal cortex | 1.40 | 10.50 | 13.20 |
| Right middle temporal gyrus | 2.00 | 10.80 | 14.90 |
| Right nucleus accumbens | 3.40 | 15.20 | 20.50 |
| Right pallidum | 1.50 | 9.50 | 12.40 |
| Right paracentral lobule | 2.00 | 12.80 | 17.30 |
| Right parahippocampal gyrus | 2.90 | 13.30 | 17.30 |
| Right pars opercularis | 3.30 | 15.70 | 20.60 |
| Right pars orbitalis | 2.90 | 14.10 | 18.50 |
| Right pars triangularis | 4.00 | 17.00 | 22.80 |
| Right pericalcarine cortex | 1.50 | 21.10 | 27.30 |
| Right postcentral gyrus | 2.00 | 11.70 | 15.20 |
| Right posterior cingulate cortex | 2.00 | 13.60 | 18.50 |
| Right precentral gyrus | 1.80 | 10.60 | 14.50 |
| Right precuneus cortex | 1.50 | 10.00 | 12.40 |
| Right putamen | 2.20 | 9.90 | 13.40 |
| Right rostral anterior cingulate cortex | 1.90 | 20.00 | 26.80 |
| Right rostral middle frontal gyrus | 3.90 | 14.20 | 20.60 |
| Right superior frontal gyrus | 2.60 | 11.00 | 15.40 |
| Right superior parietal cortex | 2.10 | 11.70 | 15.80 |
| Right superior temporal gyrus | 2.10 | 11.00 | 14.40 |
| Right supramarginal gyrus | 1.70 | 12.70 | 17.00 |
| Right temporal pole | 2.60 | 14.40 | 19.10 |
| Right thalamus proper | 2.80 | 7.80 | 10.10 |
| Right transverse temporal cortex | 2.10 | 16.40 | 21.70 |
| Right ventral diencephalon | 1.50 | 8.00 | 10.80 |
| Third ventricle | 13.00 | 38.50 | 48.70 |
